# Study of a Conformational Equilibrium of Lisinopril by HPLC, NMR, and DFT

**DOI:** 10.1155/2014/494719

**Published:** 2014-02-25

**Authors:** Sondes Bouabdallah, Med Thaieb Ben Dhia, Med Rida Driss

**Affiliations:** ^1^Laboratoire de Chimie Analytique Appliquée, Faculté des Sciences de Bizerte, 7021 Zarzouna, Tunisia; ^2^Laboratoire de Chimie Organique Structurale, Synthèse et Etude Physico-Chimique (Equipe de Chimie de Coordination), Faculté des Sciences de Tunis, 1060 Tunis, Tunisia

## Abstract

The isomerization of lisinopril has been investigated using chromatographic, NMR spectroscopic, and theoretical calculations. The NMR data, particularly the NOEDIFF experiments, show that the major species that was eluted first is the *trans* form. The proportion was 77% and 23% for the *trans* and *cis*, respectively. The thermodynamic parameters (Δ*H*, Δ*S*, and Δ*G*) were determined by varying the temperature in the NMR experiments. The interpretations of the experimental data were further supported by DFT/B3LYP calculations.

## 1. Introduction

**Figure 1:**
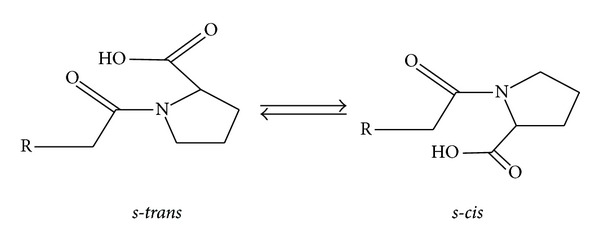
Interconversion of L-proline.

Lisinopril, N-(1-carboxy-3-phenylpropyl)-L-lysyl-L-proline, belongs to a class of antihypertensive agents which inhibit the angiotensin-converting enzyme (ACE) to control blood pressure [1]. The active parts of ACE inhibitors are peptide derivatives containing C-terminal proline residues. Like other proline-containing peptides, lisinopril exists as an equilibrium mixture of *cis* and *trans* isomers, with respect to the proline amide bond (Figure 1) [2, 3]. Under unstrained conditions most peptide bonds adopt the *trans* isomeric form, mainly because of the weaker steric repulsion between hydroxyl and carboxyl group effects in the molecule when compared to the *cis*. The *trans* form in lisinopril was shown to be the preferred isomer and biologically active [4–6]. The assignment separation of *cis* and *trans* form of lisinopril has been carried out by HPLC [5, 7–12], CZE [13–16], and NMR spectroscopy [2, 17–22]. The latter technique is a powerful tool and has been widely applied for structural and stereochemical characterization of amino acids, oligo- and polypeptides [23–26]. The *cis*-*trans* isomerization of peptide bonds is a slow process on the NMR time scale under normal conditions at ambient temperature due to the high barrier resulting from the C–N partial double bond character. NMR spectroscopy has therefore been successfully used to study the *cis-trans* isomerization process of lisinopril in solution [25, 27].

In this paper, we report on the isomerization of lisinopril using a combination of HPLC, NMR spectroscopy, and theoretical approaches. The effect of temperature on the *cis-trans* isomerization process of lisinopril was investigated in order to determine different thermodynamic parameters (Δ*H*, Δ*S*, and Δ*G*).

## 2. Experimental

### 2.1. Samples

Lisinopril was kindly provided from Solvay Pharmaceuticals.

### 2.2. Reagents

Potassium dihydrogen phosphate, sodium hydroxide, and phosphoric acid were of RP quality from Prolabo (France). Methanol, acetonitrile, and tetrahydrofuran (THF) were of HPLC grade from LabScan (Dublin, Ireland).

The mobile phase was prepared by first preparing a solution of 0.02 M KH_2_PO_4_, adjusting its pH to 2 with phosphoric acid and finally mixing the solution with an organic modifier (acetonitrile, methanol, and THF). The mobile phases were always filtered using 0.45 *μ*m membrane filter (Supelco) and degassed by sonication.

### 2.3. Chromatography

Liquid chromatographic analyses were performed using a Hewlett Packard 1100 HPLC system equipped with a photo diode array UV detector set at 215 nm. Injection was performed using an autoinjector. A Supelco LC 18 (5 *μ*m) column (250 × 4.6 mm I.D) and a guard column (20 × 4.6 I.D) both from Supelco (Bellefonte, PA, USA) were used. The pH of mobile phase buffers was adjusted by means of a Schott model CG 825 pH meter (Germany).

### 2.4. Nuclear Magnetic Resonance (NMR Spectroscopy)

^1^H NMR spectra were obtained at 300.13 MHz on a Bruker Avance III spectrometer. The probe temperature was 298 K. ^1^H chemical shifts were measured relative to tetramethylsilane [TMS, (CH_3_)_4_Si]. Spectral width was 4201.68 Hz, acquisition time 2.818 s, numbers of scans 120, FID: TD 16384, SI 16384, LB 0.100, and relaxation time was 0.5 s.

All measurements were made on lisinopril in CD_3_CN/D_2_O (1/9) solution. The variable temperature NMR spectra were acquired using the same instrument. Probe temperatures (±0.5 K) were measured with a calibrated digital thermocouple. Samples were allowed to equilibrate for 10 min at each temperature before recording the spectrum.

### 2.5. Computational Details

Density functional theory (DFT) calculations were carried out on the *cis* and *trans* isomers of lisinopril with the Gaussian 03W suite of programs [28], with the nonlocal hybrid functional denoted as B3LYP [29]. Then basis sets used were zeta 6-31+G* [30–34], doubly polarized with diffuse functions on all the atoms. The geometries of both the *cis* and *trans* isomers were optimized using an analytical gradient. The harmonic vibration frequencies of the different stationary points of the potential energy surfaces (PES) have been calculated at the same level of theory in order to identify the local minima as well as to estimate a corresponding zero-point vibrational energy (ZPE).

## 3. Results and Discussion

### 3.1. HPLC Study

The study of the *cis/trans* equilibrium of lisinopril by HPLC demonstrates that chromatographic conditions such as flow rate, temperature, pH, and organic modifier have an important effect on peak shape and retention time of lisinopril.

**Figure 2:**
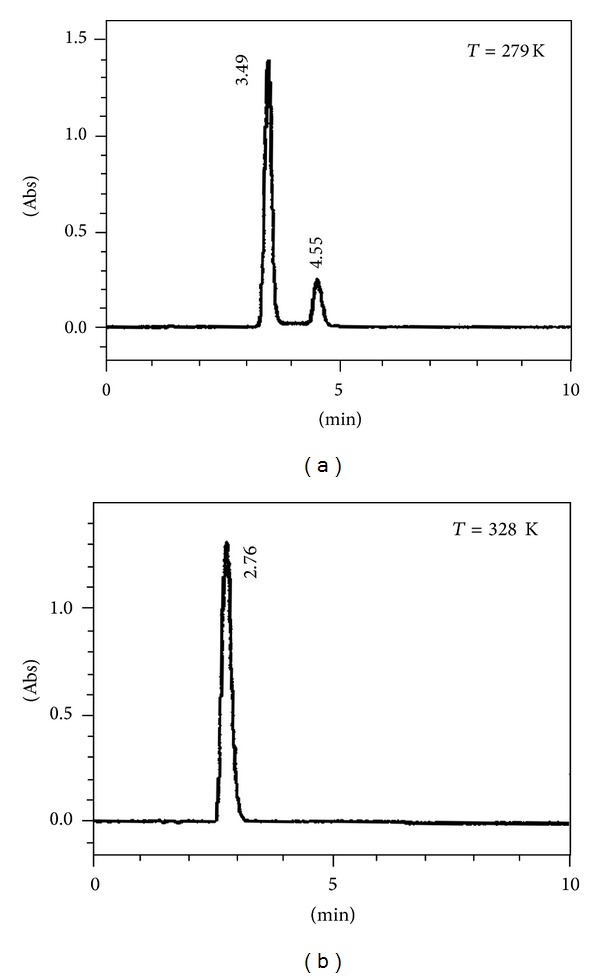
Effect of column temperature on the peak shape of lisinopril; mobile phase: phosphate buffer, [pH 7]/acetonitrile (90 : 10, v/v); flow rate: 2.0 mL/min; stationary phase: Supelco LC18, 5 *μ*m (250 × 4.6 mm I.D.). (a) *T* = 279 K, (b) *T* = 328 K.

It appears that the separation of the two isomers of lisinopril can be achieved using a mobile phase consisting of a mixture of 20 mM phosphate buffer [pH 7]-acetonitrile (90/10; v/v), a column temperature of 279 K, and flow rate of 2 mL/min with retention time *t*_*R*1_ = 3.49 min and *t*_*R*2_ = 4.55 min. However, a higher temperature is required for the elution of lisinopril as a single sharp peak at 2.76 min (Figure 2).

This is because it was found that an elevated temperature led to deterioration in the separation of the two isomers.

Moreover, at 328 K lisinopril was eluted as a narrow single peak due to the high isomerization rate of the two isomers. On the other hand, at low temperature the two isomers were resolved almost completely indicating that the interconversion rate had slowed down.

From ambient temperature chromatograms, the isomer *trans/cis* ratio was integrated to be 76/24. This result is similar to those reported earlier demonstrating that high temperature was useful for elution of proline-containing substances as a single peak [7, 11, 35]. Conversely, a low temperature is known to have a potential effect on the separation of isomers [5, 9, 36–39].

### 3.2. NMR Studies

**Figure 3:**
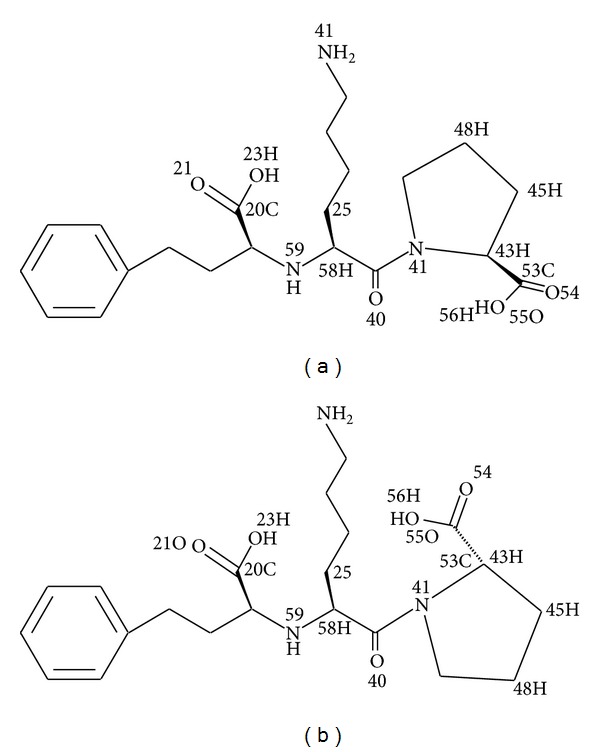
Chemical structure of lisinopril (S, S, S): (a) *cis* form and (b) *trans* form.

**Figure 4:**
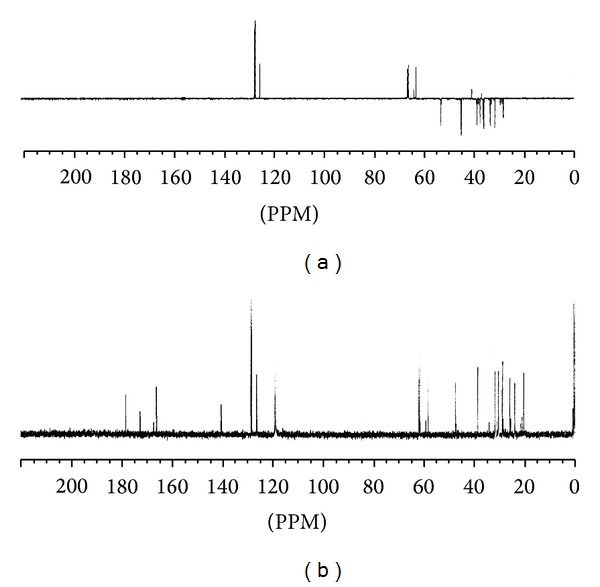
^13^C NMR spectrum of lisinopril in D_2_O/CD_3_CN (9/1, v/v). (a) Dep 135*t*, (b).

The structure of lisinopril (Figure 3) shows 21 carbon atoms with two sets of two chemically equivalent carbons describing the ortho- and metapositions on the aromatic ring. So, we expect to observe 19 signals in ^13^C NMR spectra. However, the obtained spectra showed the doubling of all signals confirming the existence of the two isomers (Figure 4).

**Figure 5:**
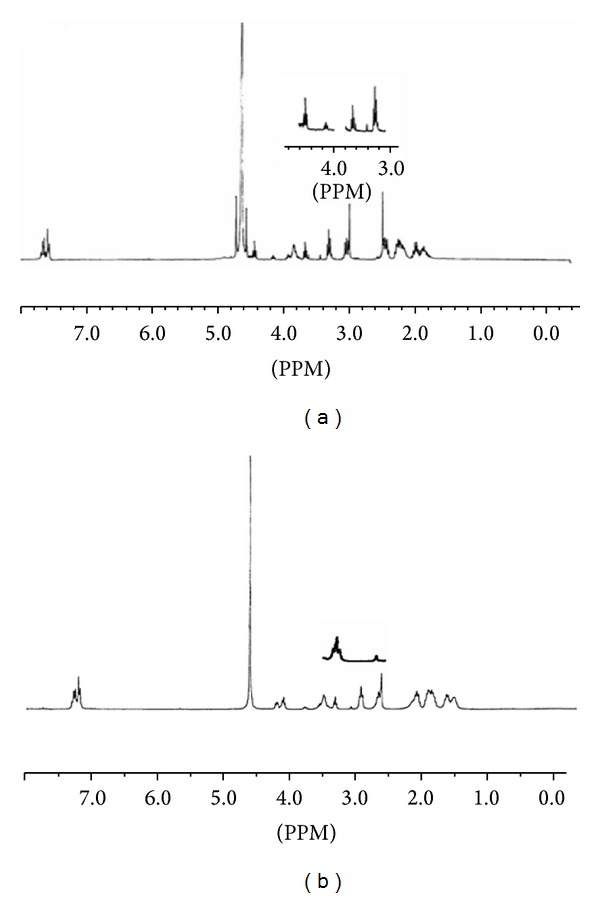
^1^H NMR spectrum of lisinopril in D_2_O/CD_3_CN (9/1, v/v). (a) *T* = 298 K, (b) *T* = 333 K.

In addition, the ^1^H NMR spectra of lisinopril in CD_3_CN/D_2_O at 298 K (Figure 5(a)) show two sets of triplets of unequal intensities. The multiplicity of each signal set reflected first from the interaction of H58 with H25 and H26, giving the two signals in the 3.8–4.1 ppm region, and second from the interaction of H43 with H45 in the 4.1–4.4 ppm region. The same spectrum recorded at 333 K (Figure 5(b)) shows a better separation of the two signals at 4.1 ppm.

These isomers are assigned to a *cis-trans* equilibrium of the rotation around the amide bond. As described earlier, it is worth noting that this equilibrium appears to be slow on the NMR time scale at ambient temperatures [37, 40]. Using the area of resonance signals of proton 58 (3.8–4.4 ppm), the isomer ratio was integrated to be 77/23 at 298 K. The result obtained in a separate experiment recorded at a probe temperature of 298 K is consistent with that determined by HPLC at the same temperature.

We conclude that the major conformer in the ^1^H NMR spectrum of lisinopril corresponds to the first eluted peak in the HPLC chromatogram at ambient temperature, which exists in a higher proportion. A similar study demonstrated this correspondence in the case of ramiprilat [4], enalaprilat [5], and perindopril [39] in different solvents.

It is well known that the NOE effect (*ζ*) between two dipole-dipole interacting nuclei is inversely proportional to the distance (*r*_*ij*_)^6^ between the irradiation site (*i*) and the measured one (*j*), respectively [41], according to the following formula: *ξ*_*i*_(*s*) = *f*(1/*r*_*is*_^6^).

Therefore, a low NOE (*ζ* = 8%) is observed at H_58_ (*δ* = 3.16 ppm) when H_43_ (*δ* = 4.35 ppm) is irradiated in the major conformer. Accordingly, a stronger NOE (*ζ* = 21%) is noted at H_58_ (*δ* = 3.7 ppm) when H_43_ (*δ* = 3.31 ppm) is irradiated in the minor conformer.

**Figure 6:**
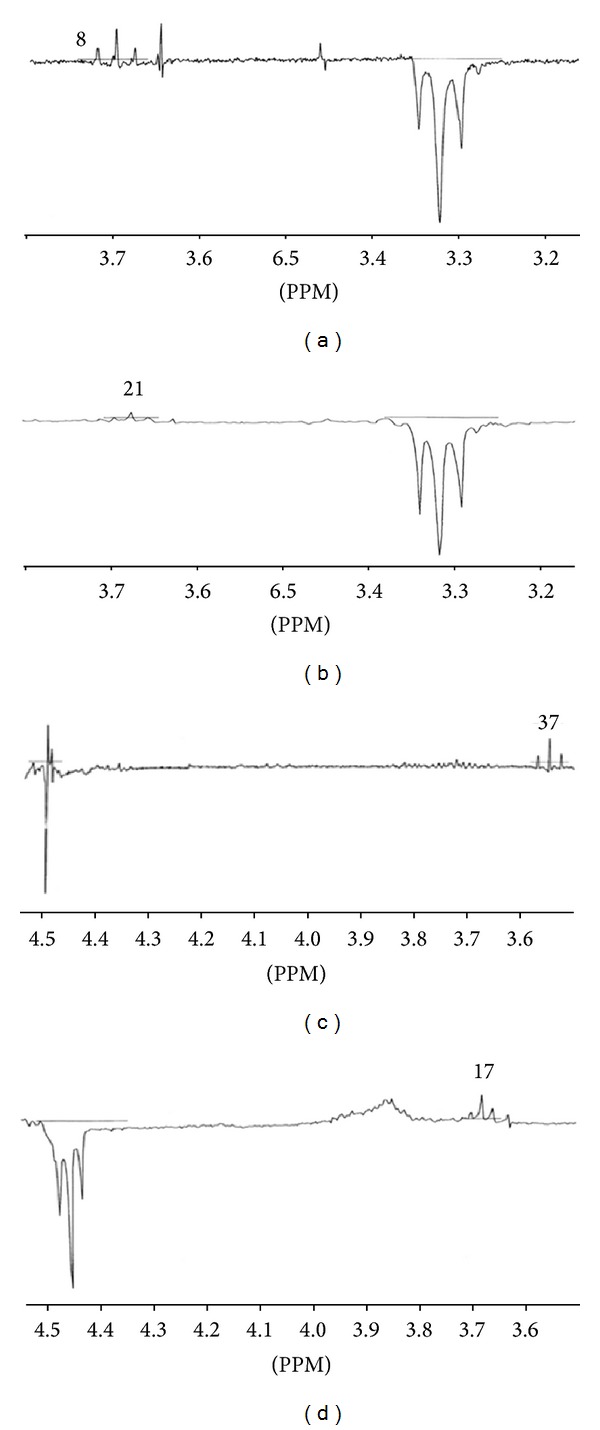
NOE difference spectra of lisinopril in D_2_O/CD_3_CN (9/1, v/v), at 298 K and 300 MHz. Irradiation of H43 and H48 (a, c) major conformer; (b, d) minor conformer. The numbers next to the peaks represent the quantified NOE at H58.

In addition, when H_48_ (*δ* = 4.45 ppm) is irradiated, we observe at H_58_ a NOE (*ζ* = 34%) in the major conformer and a NOE (*ζ* = 10%) in the minor conformer (Figure 6).

This shows that the NOE in the minor conformer for the H43/H58 is more important than the NOE in the major conformer, which implies that in the major conformer, the distance between the nuclei is higher than the one in the minor conformer

(1) $$\begin{array}{c} r_{43\text{–}58(\min)}<r_{43\text{–}58(\text{maj})},\\ r_{48\text{–}58(\text{maj})}<r_{48\text{–}58(\min)}. \end{array}$$

Based on the relationship between NOE and internuclear distances, one can give the expression of distance in each conformer

(2) $$\begin{array}{c} {\left(r_{43\text{–}58}\right)}_{\min}={\left(\frac{8}{21}\right)}^{1/6}{\left(r_{43\text{–}58}\right)}_{\text{maj}},\\ {\left(r_{48\text{–}58}\right)}_{\min}={\left(\frac{34}{10}\right)}^{1/6}{\left(r_{48\text{–}58}\right)}_{\text{maj}}. \end{array}$$

Consequently, the examination of the molecular structure of each conformer of lisinopril confirmed that the distance *r*_43–58_ in the *cis* conformer is indeed smaller than the distance in the *trans* form. The ^1^H NMR intensities suggest that the major conformer is the *trans* form and the minor conformer is the *cis* form. The nuclei of the *s-trans* conformer are more deshielded than those of *s-cis* conformer, in agreement with literature results [41, 42].

### 3.3. Thermodynamic Study

At slow chemical exchange, the relative proportion of the two conformers at different temperatures in the range (279–333) K and the *cis/trans* equilibrium constant of lisinopril have been measured by relative integrals of the two resonance signals of the two states of isomerizations of lisinopril.

These signals are well resolved and allowed to determine accurately the equilibrium constants for the *cis* to *trans* interconversion at different temperatures and to measure the thermodynamic parameters: enthalpy (Δ*H*°), entropy (Δ*S*°) of the equilibrium on the basis of the van't Hoff equation. The Gibbs free enthalpy (Δ*G*°) is deduced at ambient temperature.

**Figure 7:**
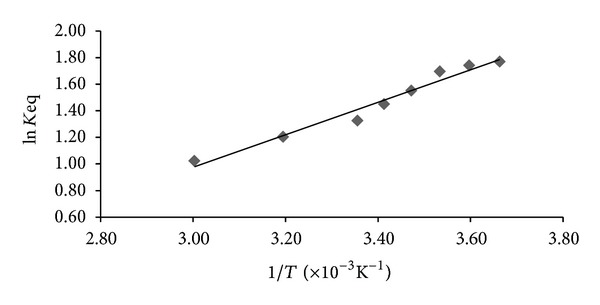
Representative van't Hoff plot in D_2_O/ACND_3_ (9/1).

The plot of ln⁡*K* versus the reciprocal of the absolute temperature is a straight line of equation ln *K* = 1212.91/*T* − 2.6359 (Figure 7). The correlation coefficient for this straight line is *r* = 0.995. The enthalpy was obtained via the slope and the entropy via the intercept of plot. The thermodynamic parameters obtained from experiment were Δ*H*° = −10.36 kJ/mol, Δ*S*° = 21.91 J/k·mol, and Δ*G*_298 K_ = −16.91 kJ/mol.

Remarkably, the equilibrium *cis* to *trans* isomerization was enthalpically and entropically favored in this condition. Consequently, the decrease in the temperature expected a displacement of the conformational equilibrium to the *s-trans* conformer. The latter is stabilized by hydrogen bonding between the carbonyl (40) and the hydrogen of hydroxyl group OH of the acid function (56). This result is in agreement with other studies reported on a similar product such as enalapril [5].

### 3.4. Theoretical Calculations

**Figure 8:**
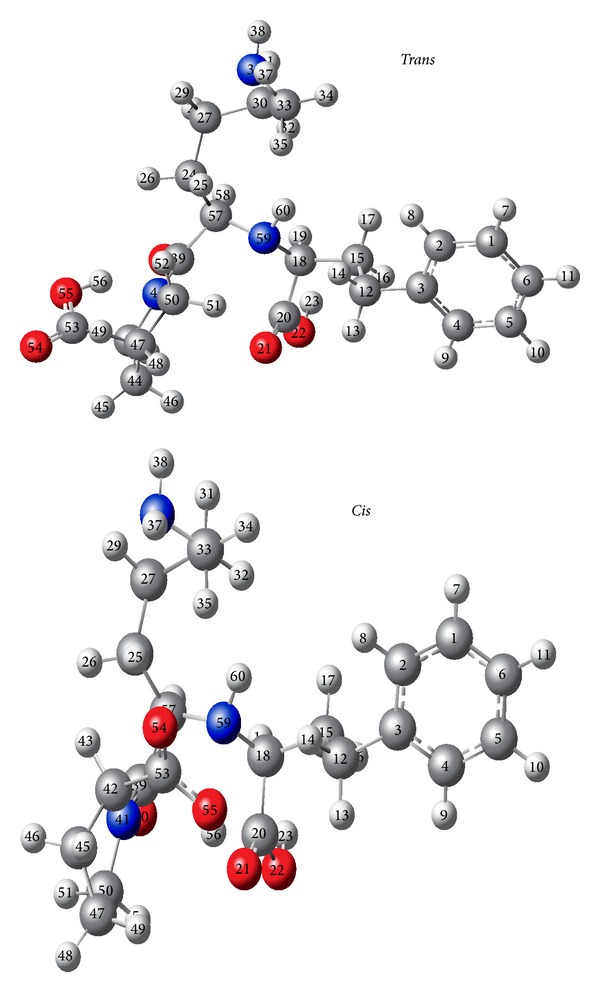
DFT/B3LYP optimized geometries of lisinopril.

In order to confirm the NMR data obtained for the *cis/trans* isomerization of lisinopril, the geometrics of the two conformers were fully optimized at the DFT/B3LYP level of theory using 6-31++G* basis set. The structures have been identified as local minima on the singlet potential energy surfaces (PES) (Figure 8). Optimized values of selected geometrical parameters are listed in Table 1. The potential energy difference between the two isomers of lisinopril was 11.397 kJ/mol indicating the stability of *trans* over the *cis* isomer. This difference is in good agreement with experimental data that the *trans* is the majorities form.

As shown in Table 1, in the *trans* conformer the interaction between H56-O40 (*d*_H56-O40_ = 1.717 Å) would be stronger than that in the *cis* (*d*_H56-O40_ = 3.857 Å), thus generating a sharp reduction of the valence angle C53-O55-H56 (*θ* = 103.1° in the *trans* form and *θ* = 2.3° in the *cis* form) and a strong variation on dihedral angle O40-C39-C41-C50 (*ϕ* = 176.6° in the *trans* form and 2.6° in the *cis* form), indicating possible existence of some hydrogen bond interactions which would be more favored in the *trans* than in *cis* isomers.

This stability of *trans* form over the *cis* form is further confirmed by the charge density between the same atoms (Δ*q*_*s*-*trans*_ = 0.08, Δ*q*_*s*-*cis*_ = 0.02). The strong interaction between O21-H56 atoms (*d*_H56-O21_ = 1.799 Å) in the *s-cis* conformer triggers a modification of the dihedral angle O21-H56-O55-C53 (*ϕ* = 156.8°) which may explain the low stability of the *cis* form. On the other hand, the low interaction between H56-O21 (*d*_H56-O21_ = 4.816 Å) in the *s-trans* conformer yields a change in the dihedral angle (*ϕ* = 3.9) so the atoms H56 and O40 were far from each other and the *trans* was more stable.

It is worth noting that there are two hydrogen bonds, one between atoms H56-O40 and another between H55-O21, but the stability was determined by the first bond because the distance is shorter (*d*_O21-H56_ = 1.799 Å). In addition, there are strong intramolecular interactions in the *trans* conformer (*μ* = 10.6 D) than in the *cis* form (*μ* = 3.9 D) indicating a higher stability of the conformer *trans*.

The distance between the nuclei in both conformers (*cis* and *trans*) was compared. It is revealed that in the *cis* conformer, H43 is close to H58 (4.058 Å), while H48 is at a much greater distance from H58 (6.612 Å). On the other hand, in the *trans* conformer H43 is further away from H58 (4.471 Å), while H48 is at much greater distance from H58 (6.225 Å). This is consistent with the results obtained with NOE difference experiments giving an enhancement of 8% and 21% in the first irradiation and 34% and 10% in the second irradiation.

## 4. Conclusions

Lisinopril exists individually as a mixture of *cis-trans* isomers in solution. The two isomers could be easily distinguished by HPLC, ^H^NMR, ^C^NMR, and ^1^H-^1^H NOE spectra. The results of the various investigations by NMR and those of the theoretical study are in favor of assigning *trans* form to the major isomer present under the conditions used in the HPLC studies. Additionally, a relative to successful combination of molecular modeling studies with experimental spectroscopic assays was used in order to elucidate the molecular bases.

## Figures and Tables

**Table 1:** Selected bond lengths (Å) and angles (deg) for the *cis* and *trans* isomer of lisinopril.

Parameter	*s-cis *	*s-trans *
Lengths		
H56–O40	3.857	1.717
H56–O21	1.799	4.816
H23–O40	3.618	4.033
H56–N41	2.486	2.613
H56–N59	2.660	4.875
H23–N59	3.721	3.717
H23–N41	4.958	4.947

Bonds lengths		
H56–O55	0.988	0.995
O55–C53	1.338	1.338
H23–O22	0.972	0.972
O22–C20	1.346	1.556
H43–H58	**4.058**	**4.471**
H48–H58	**6.612**	**6.255**
H49–H58	5.982	5.934
Energy (au)	−1359.2686	−1359.2729
Energy (Kcal/mol)	−852953.828	−852956.552
µ (Debye)	3.9300	10.6461

Bond angle		
C53–O55–H56	112.8	103.1
C18–C20–O21	125.0	124.8
N41–C39–O40	120.5	121.2
O54–C53–O56	121.6	122.2

Dihedral angle		
H23–O22–C23–O24	172.5	174.2
O40–C39–C41–C50	2.3	176.6
O54–C53–O55–C53	−158.2	178.1
O40–H56–O55–C53	7.7	−41.8
O21–H56–O55–C53	156.8	3.9

Charge		
O40	−0.49	−0.53
H56	+0.47	+0.45
O55	−0.60	−0.50
H23	+0.42	+0.42
O22	−0.55	−0.56
